# Evaluation of a surgical task sharing training programme’s logbook system in Sierra Leone

**DOI:** 10.1186/s12909-019-1647-2

**Published:** 2019-06-11

**Authors:** Ø. V. Svendsen, C. Helgerud, A. J. van Duinen, Ø. Salvesen, P. M. George, H. A. Bolkan

**Affiliations:** 1 Department of Clinical and Molecular Medicine, Norwegian University of Science and Technology (NTNU), P.O. Box 8905 MTFS, 7491 Trondheim, Norway; 2 Clinic of Surgery, St. Olavs Hospital, P.O. Box 3250 Sluppen, 7006 Trondheim, Norway; 3 CapaCare, c/o Dr Håkon Bolkan, Clinic of Surgery, St. Olavs Hospital, P.O. Box 3250 Sluppen, 7006 Trondheim, Norway; 4 Faculty Administration, Faculty of Medicine and Health Sciences, Norwegian University of Science and Technology (NTNU), 7491 Trondheim, Norway; 5 Surgical Department, University of Sierra Leone Teaching Hospital, Freetown, Sierra Leone

**Keywords:** Training, Education, Global surgery

## Abstract

**Background:**

Personal logbooks are universally applied for monitoring and evaluation of surgical trainees; however, the quality and accuracy of such logbooks in low income countries (LICs) are poorly examined. Logbooks are kept by the individual trainee and detail every surgical procedure they perform and their role during the procedure. The aim of this study was to evaluate the quality of such a logbook system in Sierra Leone and to identify areas of improvement.

**Methods:**

The last 100 logbook entries for students and graduates participating in a surgical task sharing training programme were compared with hospital records (HRs). The logbook entries were categorized as *matching*, *close matching* or *over-reported*. Moreover, HRs were checked for *under-reported* procedures. Semi-structured interviews were conducted with the study participants on logbook recording routines. The results were analysed using mixed effects logistic regression models.

**Results:**

Three thousand one hundred sixty-nine database entries from 35 participants were analysed. Of that amount, 62.2% of the entries matched the HRs, 10.4% were close matches and 26.9% were over-reported. 20.7% of the investigated HRs were under-reported.

**Conclusions:**

Information gathered from surgical logbook systems must be applied with care, and great efforts must be made to ensure that the logbook systems used provide reliable data. Based on analysis of the logbook data and interviews, focus areas are suggested to ensure reliable logbook data in LICs. Clear instructions and proper training should be provided when introducing the logbook system to the users. The importance of logging all procedures, including minor ones, should be emphasized. The logbook system should be user friendly and only as extensive as necessary. Lastly, keeping the logbooks exclusively digital is recommended, combined with sufficient IT equipment and training.

**Electronic supplementary material:**

The online version of this article (10.1186/s12909-019-1647-2) contains supplementary material, which is available to authorized users.

## Background

It is estimated that 4.8 billion people, i.e. 67% of the world’s population, lack access to even the most basic surgical care [1]. Only 6% of the 313 million surgical procedures annually performed worldwide occur in the poorest countries, and 143 million additional procedures per year are required to cover the unmet needs of low and middle income countries [2].

One of the main barriers to the expansion of surgical capacity in low income countries (LICs) is the shortage of human resources [3]. New and innovative models of training are needed to meet the demand for qualified personnel, and surgical task sharing is a strategy increasingly applied to address this issue [4]. Task sharing is defined as transferring tasks from one professional to another for more efficient use of scarce human resources [2]. Applied in the surgical field, this means training non-specialists to be able to perform certain basic surgical and obstetric procedures, e.g. hernia repairs, caesarean sections, appendectomies and more. However, there is little knowledge on how to optimally provide effective and efficient surgical training. Especially in LICs, where trainer resources are limited, monitoring the progress during the training in these programmes is, therefore, of great importance to assure that the trainees gain the required skills and experience [5].

Surgical logbooks are tools commonly applied for monitoring and evaluating surgical trainees’ progress [6]. Logbooks are kept by the individual trainee and detail every surgical procedure they perform and their role during the procedure to be able to follow progress in the training programme. Few studies have examined the quality of such recordkeeping in LICs. The aim of this study was to evaluate the accuracy of self-reported surgical logbooks used in that setting, and to identify potential areas of improvement.

## Methods

### Setting

Sierra Leone is an LIC where less than 8% of the population’s perceived need for surgery was met in 2012 [7]. An estimated 25% of the annual deaths in the country might have been prevented with sufficient access to surgical services [8].

The Sierra Leonean Ministry of Health and Sanitation has been working in cooperation with the non-governmental organization CapaCare since 2011 to improve capacity in basic life-saving surgery and obstetrics through task sharing [9]. This is achieved by enrolling medical doctors and community health officers into a surgical training programme, training the participants to manage emergency surgical and obstetric pathologies. The first 2 years entail practical and theoretical surgical training, along with clinical rotations within a consortium of training hospitals, followed by a one-year internship at the main surgical and obstetrical teaching hospitals in the capital, Freetown [10]. Graduates from the programme are posted in hospitals throughout the country. For the remainder of this article, *students* refers to participants in the first 2 years of training, *interns* refers to participants during the one-year internship, while *graduates* refers to participants that have completed both the first 2 years of training and the internship year of the programme.

Students, interns and graduates keep identical handwritten personal logbooks (PLs). The PLs are used by the programme administration for monitoring and evaluation of the progress and surgical exposure of students and interns, programme reports on activity and impact, as well as implementation research. They contain information on the date of the operation, hospital, patient sex and age, preoperative diagnosis, type of procedure and outcome. All procedures entered in the PLs, either performed or observed are to be signed by a clinical supervisor. Every month, students, interns and graduates transfer their PL into an Excel document and submit to a central database containing all generated entries. By 1 July 2016, 27,219 entries had been recorded in the database since the initiation of the programme in 2011 [9].

### Selection and data collection

By July 2016, 12 graduates had completed the training, while 24 students and interns were currently enrolled in the programme. All 36 students, interns and graduates were eligible for this study. For each participant, the last 100 database entries obtained before 1 July 2016 were included in the study, regardless of the time span these 100 procedures covered. For participants with less than 100 entries before this date, all entries were included.

Fifth year medical students from the Norwegian University of Science and Technology collected data for this study between September 7, 2016 and October 17, 2016. Hospital records (HRs) were collected from all 19 hospitals where procedures in the selection had been performed. The HRs consisted of handwritten operation theatre books or anaesthesia books or both, which were photographed at the hospitals for later cross-checking with the database. Pictures of the participants’ PLs were also taken for later cross-checking with the database. Semi-structured interviews were conducted with the study participants about their routines for recording the surgical data (Additional file 1).

### Data analysis

Four criteria were applied to check the accuracy of the database entries, using the HRs as a gold standard; date of operation, patient ID, type of procedure and name of the study participant (Table 1). A database entry was considered a match if all four criteria were met. A close match was allowed failure to satisfy one of the first three criteria. Close matched entries were further subcategorized according to which of the three criteria was erroneous. Entries in the PL not satisfying the criteria for match or close match were considered over-reported.

**Table 1:** Match criteria

1: Identical date of operation (+/− one day)	
2: Identical patient ID (two out of three)	
2.1 Initials	
2.2 Sex	
2.3 Age	
3: Identical procedure	
4: Name of the study participant recorded in the hospital record entry (not required if student role was “observing”)	

For each participant, HRs were examined in the period ranging from the first database entry in the participant’s selection to 1 July 2016. Any procedure containing the participant’s name that was not registered in the database was considered under-reported. To get more information about where discrepancies occur, we also compared the selected entries in the database to the PLs. More information about how specific cases were handled in the cross-matching process can be found in Additional file 2.

### Statistics

The logbook entries were grouped by the progress of the participant in the programme (student, intern or graduate) and procedure extent (definition minor or major surgery, see Additional file 3). This made it possible to better understand the causes of discrepancy between the datasets.

We calculated point estimates of the percentages of entries in the database that were matching, close matching and over-reported. The percentage of under-reported procedures was calculated as the percentage of HR entries that was not registered in the database. To calculate the 95% confidence intervals, mixed effects logistic regression models with the Laplace approximation were used, where participant ID and hospital ID were specified as random effects. Likelihood ratio tests for procedure extent and participant progress were performed, and where significant, pairwise comparisons against a reference category were done. The significance threshold was set at 0.05. Stata/IC version 13.1 was used for all statistical analysis.

## Results

**Fig. 1:**
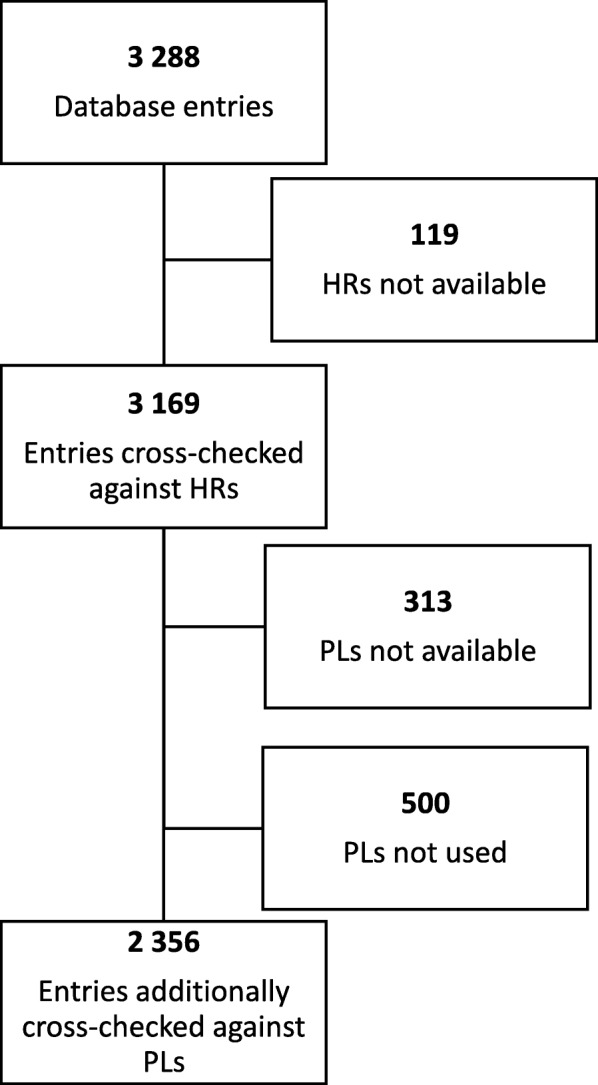
Selection and exclusion of database entries

From 3288 eligible entries obtained from the database, 3169 (96.4%) were included in the final analysis (Fig. 1). All 100 entries from one graduate performed at a hospital were excluded because no HRs were available. Nineteen additional entries from two other participants at another hospital were excluded because parts of the HRs were missing and matching was not possible. Entries from a total of 35 participants—18 students, 6 interns and 11 graduates at 18 different hospitals—were available for analysis.

Of the 3169 database entries cross-checked against the HRs, 813 were missing associated PL entries. The remaining 2356 (74.3%) were cross-checked against the PLs in addition to the HRs. Interviews were performed with all participants but one. The results from the interviews can be found in Additional file 1.

When cross-checking the entries in the database with the HRs, a match percentage of 62.6% (95% CI 45–77, *n* = 1984), close matches in 10.5% (95% CI 7–15, *n* = 333) and over-reporting in 26.9% (95% CI 15–44, *n* = 852) of the entries was found. 20.7% (95% CI 14–30, *n* = 656) of the procedures in the HRs were under-reported (Table 2). Combining all matches and close matches results in a total of 73.1% (95% CI 56–85, *n* = 2317) of the database entries identified in HRs.

**Table 2:** Results from cross-checking the database against the HRs

	Percentage	Match % (95% CI)	Close match % (95% CI)	Over-reporting % (95% CI)	Under-reporting % (95% CI)
Total (*n* = 3169)	100	62.6% (45–77)	10.5% (7–15)	26.9% (15–44)	20.7% (14–30)
Procedure extent					
Major^a^ (*n* = 2589)	81.7	65.5% (48–80)	10.5% (7–15)	24.0% (13–41)	18.5% (12–28)
Minor (*n* = 454)	14.3	51.1%^**^ (33–69)	7.5% (5–12)	41.4%^**^ (24–61)	33.7%^**^ (22–48)
Unspecified (*n* = 126)	4.0	44.4%^**^ (26–65)	7.5%^*^ (4–14)	35.7%^*^ (19–58)	15.6%^**^ (8–28)
Participant progress					
Graduate^a^ (*n* = 701)	22.1	78.9% (57–91)	12.7% (7–22)	8.4% (3–21)	21.2% (11–36)
Internship (*n* = 818)	25.8	50.1% (22–79)	10.0%^*^ (4–23)	39.9%^**^ (16–70)	29.0%^*^ (13–54)
Student (*n* = 1650)	52.0	61.8% (43–78)	9.8%^*^ (6–16)	28.4%^*^ (15–46)	16.4% (9–28)

^a^Reference category

^*^Significantly different from reference category (*p* < 0.05)

^**^ Significantly different from reference category (*p* < 0.01)

**Fig. 2:**
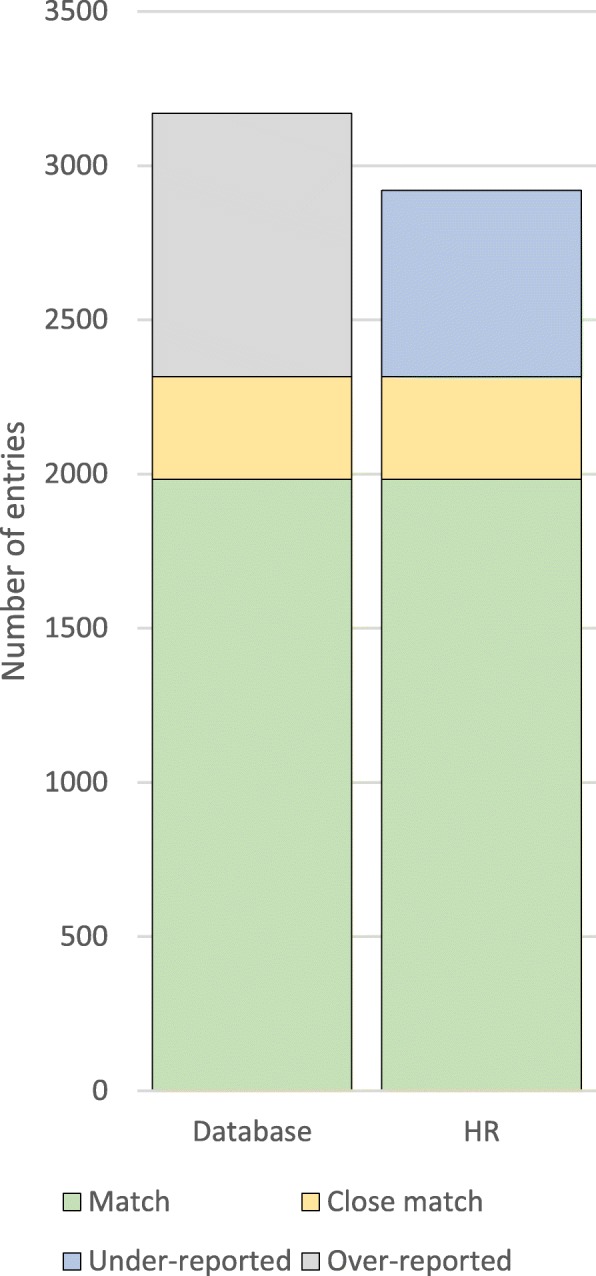
Comparison of over- and under-reporting

The number of selected entries in the database was 8.5% higher than the number of procedures found in the HRs, due to more over-reported than under-reported entries (Fig. 2). In other words, the net over-reporting is 8.5%. The distribution of minor and major procedures was the same among the under-reported and over-reported procedures, with 76.8% of the entries in both groups being major and 23.2% being minor procedures. Out of all minor procedures in the HRs, one-third was under-reported. In contrast, this number was 18.5% for the major procedures.

**Fig. 3:**
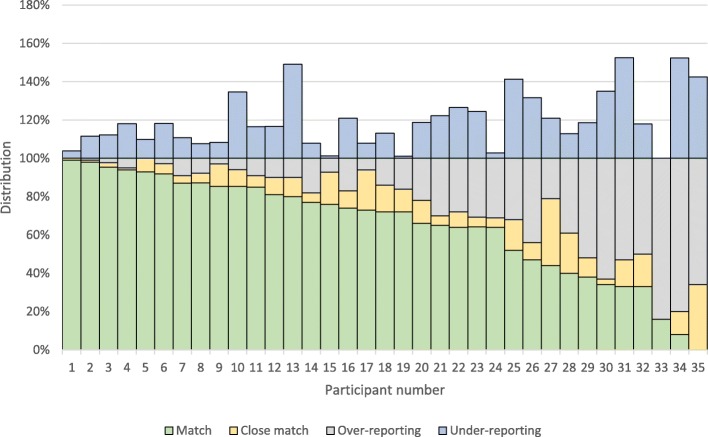
Distribution of match, close match, over- and under-reporting for each individual participant

The match percentages of individual participants ranged from 0 to 99% (Fig. 3). The match percentages of students and interns were not significantly different from the graduates (Table 2). A significantly lower percentage of over-reporting (*p* < 0.001) and under-reporting (*p* < 0.001) of major procedures was found compared to minor procedures.

### Close matches between database and HRs

Incorrect patient ID contributed most to the close matches with 53.6% (95% CI 32–74, *n* = 178), followed by incorrect procedure with 37.7% (95% CI 18–63, *n* = 125) and incorrect date with 8.7% (95% CI 4–18, *n* = 29).

### The database and PLs compared

Ninety per cent (95% CI 81–95, *n* = 2135) of the database entries matched the PLs, while 6.5% (95% CI 3–13, *n* = 154) of the procedures were close matches and 3.5% (95% CI 2–8, *n* = 84) of the database entries were not found in the PLs. Finally, 3.1% (95% CI 1–7, *n* = 73) of the PL entries were not found in the database.

## Discussion

When looking at the overall distribution of match (62.6%), close match (10.5%) and over-reporting (26.9%) among the selected entries in the database, the logbook system shows room for improvement. The proportion of under-reported procedures in the HRs (20.7%) further emphasizes this. However, two observations considerably improve this picture.

Firstly, the majority of the close matched entries may be regarded as high enough quality to be valid. The entries containing incorrect date or patient ID contributed to 62.3% of all close matches. These criteria may have little or no impact on the quality of the surgical data in the database. This justifies the combination of matching and close matching entries to a total of 73.1% of the database entries identified in the HRs. This result is comparable to the logbooks of American surgical residents, where 75.1% of the entries were identified in HRs and with errors in 47.2% of the procedure codes [11]. Secondly, the net over-reporting is only 8.5%. The similarities between the over- and under-reported cases justify the combination of these groups.

More detailed analysis of the results found in the present study provides information about important focus areas to ensure that logbook systems in LICs provide even more reliable data.

The combination of clear instructions and proper training in using the logbook system would likely increase the accuracy of the data. The differences in accuracy between individual participants suggest that the cause of errors is due to individual factors to a certain extent. By ensuring all users of the logbook system have the correct understanding of proper usage, better results can be expected for the users with less accurate data. During the interviews, three participants requested better training in the usage of the logbook systems in the beginning of the programme. This is also identified in previous evaluations of electronic surgical logbooks, where clear instructions regarding data entry has been a key recommendation [12].

Emphasizing the importance of registering all procedures regardless of the severity of the procedure could reduce under-reporting, especially for minor procedures. A higher proportion of under-reporting is seen for minor procedures than for major procedures, and 10 participants reported that they often do not record certain minor procedures.

Making the logbook systems user friendly and only as extensive as necessary is important to ensure it is used as intended. Qualified health care personnel is a limited resource in most LICs [13], often making the workload on surgical trainees immense. Entry of data into logbooks can be a time-consuming process, and procedures can easily be forgotten or omitted in a hectic clinical setting. Five of the participants in our study who admitted some under-reporting in the interviews pointed out workload as one of the main reasons for this.

Making logbook systems that are exclusively digital would likely improve their accuracy, as some of the errors found in this study seem to have appeared during the transfer between the handwritten PLs and the digital database. Indeed, only 90% of the procedures in the database matched the PLs without errors. During the data analysis, it became evident that the transfer of information between different data sources is often done in bulk, making systematic errors likely. This could, at least in part, explain why the results from a few participants were close to 0% match.

Proper IT equipment and training is a necessary part of a reliable logbook system. This became evident during the interviews, where a participant reported that lack of IT skills was an issue, and nearly one-quarter of the participants reported issues with plotting the data into Excel documents. Old and low-quality IT equipment was also a common complaint that made the process more complicated for many participants. Addressing these issues might help reduce the considerable interpersonal variance in reporting quality.

### Limitations

HRs were chosen as the gold standard. Entries in anaesthesia books are normally written by the responsible anaesthesist at the procedure, while entries in the operation theatre books are normally written by one of the surgeons involved. Thus, an entry in the operation theatre book and the PL will in many cases be written by the same person. This could mean that a procedure unregistered in HRs is more likely to be unregistered in the PL, and the rate of under-reporting found in this study might therefore be too low. However, HRs from hospitals in Uganda comparable to our HRs has been shown to contain 99% of all procedures and 94% of post-operative deaths [14]. The HRs are not perfect as a gold standard, but most likely the best one available.

Difficulties regarding data collection is a well-known challenge when performing research in developing countries and often results from inadequate documentation and fragmented data [14]. The HRs investigated in this study were without exception handwritten paper books. These are fragile documents, and in many cases the documents were torn and in poor condition. On a few occasions, certain fields in the HR entries were blank. Interpretation of the handwriting was also an issue. This could cause correctly entered procedures to be classified as close matches or over-reported.

Hospitals were registered with three letter abbreviations, which may be a reason for mismatch. Incorrect date in the entries could further cause errors in the matching process, resulting in some matches and close matches possibly being registered as over-reported. Correct hospital abbreviation was not a criterion for an entry to be a match, but wrong hospital abbreviation would make it unlikely for the procedure to be found in the HRs during our matching. Incorrect date could be caused both by date approximations made by the participant and by confusion regarding the date format (Additional file 1). The entry could still be a close match, but as with the case of incorrect hospital abbreviation, the entry would be unlikely to be found in the HRs during the process of matching.

### Key recommendations

Based on the results and discussion presented in this article, the following recommendations for surgical logbook systems in LICs are advised:

- Provide clear instructions and proper training when introducing the logbook system to the users
- Emphasize the importance of logging all procedures, including minor ones
- Make the logbook system user friendly and only as extensive as necessary
- Keep the logbooks exclusively digital
- Provide sufficient IT equipment and training

## Conclusions

Monitoring and evaluation is an important part of surgical training programmes in LICs, and logbooks are useful and commonly applied tools for this purpose. After evaluating a logbook system and the accuracy of the associated database, it is clear that accurate surgical data can be achieved with such a system. However, implementing systems of satisfactory quality is challenging, especially in LICs, and great efforts must be made to ensure the data is of good quality.

## Additional files

Additional file 1: Semi-structured interviews. Summary of answers given during the semi-structured interviews conducted with the study participants. (DOCX 17 kb)

Additional file 2: Handling of specific cases. Details of how some specific cases were handled during data analysis. (DOCX 13 kb)

Additional file 3: Minor and major procedures. Description of which procedures were defined as minor and major. (DOCX 14 kb)

## Data Availability

The datasets analysed in this study contains sensitive health data and can not be made publicly available. A summary of the semi-structured interviews can be found in Additional file 1.

## Abbreviations

CI: Confidence interval
HR: Hospital record
LIC: Low income country
PL: Personal logbook
