# Supplementary material for: Evaluation of a surgical task sharing training programme’s logbook system in Sierra Leone
Source: BMC Med Educ. 2019 Jun 11;19:198. doi: 10.1186/s12909-019-1647-2 (PMC6560768; doi:10.1186/s12909-019-1647-2)
Supplement: Supplementary file 1 — Semi-structured interviews. Summary of answers given during the semi-structured interviews conducted with the study participants. (DOCX 17 kb) [file 12909_2019_1647_MOESM1_ESM.docx]

# **Additional file 1 Semi-structured interviews**

Semi-structured interviews were performed with 34 out of 35 participants. One participant was unavailable for interview. The interview consisted of both multiple-choice questions and questions open for the participants’ own formulations.

**1. What is the average time interval between performance and registration of a procedure in your logbook?**

- **Under 1 day** (20/34)
- **1–6 days** (12/34)
- **7–20 days** (2/34)
- **21–90 days** (0/34)
- **Over 90 days** (0/34)

**2. Do you have a handwritten version of the logbook that you transfer to a digital version?**

- 24 answered “yes”
- 5 reported entering the information in the digital version first and transferring it to the handwritten version later to be signed by the surgical tutor
- 5 reported using only the digital version

**3. Have you encountered any problems when entering information into the digital logbook version?**

- 12 reported problems with their tablet/computer
- 8 reported problems plotting in Excel
- 3 said lack of electricity to charge their laptop/tablet was an issue
- 1 mentioned workload as a challenge
- 1 said it was problematic that the soft copy requires the entering of a supervisor, when the participant mostly operates alone

**4. Have you encountered any problems when admitting the digital logbook to the CapaCare organization?**

- 22 said that lack of available Internet connectivity was an issue
- 3 reported problems using the mail app on their tablet
- 1 reported technical issues with the tablet when admitting the digital file

**5. How often do *you* register information about procedures you have performed in HRs?**

- When performing the procedure
  - Most of the times (29/34)
  - Sometimes (1/34)
- When assisting the procedure
  - Most of the times (6/34)
  - Sometimes (10/34)
- When observing the procedure
  - Most of the times (0/34)
  - Sometimes (1/34)
- 1 reported that it always depends on who he/she is working with

**6. During your time in CapaCare, have you attended any procedures without registering it in the written logbook? If yes, for what reasons?**

- 20 reported having under-reported some procedures during their time in CapaCare.

The following reasons were mentioned:

- Under-reporting due to the procedure being a minor one (10/34)
- Workload at the hospital (5/34)
- Not entering procedures performed when visiting hospitals other than the one the participant is posted at (3/34)
- Sometimes forgetting to log procedures (2/34)
- Sometimes when only observing the procedure (2/34)
- When a suiting procedure type is not available from the soft copy menu (2/34)
- When arriving halfway into the procedure (1/34)
- Only registers one entry when cleaning and dressing the same patient with chronic ulcer over time (1/34)

**7. During your time in CapaCare, have you registered any procedures that have not taken place? If yes, for what reasons?**

- None of the participants answered “yes” to this question

**8. Do you have any additional comments regarding the logbook system?**

- 3 participants wanted more training in using the logbook system
- 1 participant said that IT skills is an issue for many of the surgical trainees, and he/she would like training in this
- 1 candidate reported having seen some cases of mistakes in operating theatre (OT) book regarding patient name. This was reportedly because some surgeons use the consent form for filling in the patient name in the OT book, when the consent form in most cases is filled in by family members
- 1 candidate reported confusion using the 24-hour time format, in addition to the database date format (DD.MM.YY) that differs from the most common date format in Sierra Leone (MM.DD.YY)
