# Supplementary material for: Evaluation of a surgical task sharing training programme’s logbook system in Sierra Leone
Source: BMC Med Educ. 2019 Jun 11;19:198. doi: 10.1186/s12909-019-1647-2 (PMC6560768; doi:10.1186/s12909-019-1647-2)
Supplement: Supplementary file 2 — Handling of specific cases. Details of how some specific cases were handled during data analysis. (DOCX 13 kb) [file 12909_2019_1647_MOESM2_ESM.docx]

# **Additional file 2 Handling of specific cases**

One participant’s selection of procedures started in 2014. At one of the hospitals where this participant had performed procedures, the hospital records (HRs) from 2014 were not available and consequently could not be checked for under-reported procedures. According to the database, the participant only performed procedures at another hospital during this period.

An empty field in any of the data sets was considered incorrect. If multiple procedures were performed during an operation, either a single entry or several separate entries in the database was accepted.

Under-reported procedures without date listing were accepted if the closest procedure before and after were listed with a date within the participant’s selection of database entries.

Some HRs did not contain patient age or sex. When required, sex was determined from the patient’s given name or the type of procedure or both.

Given names are often used in the HRs, and some participants have the same given name. If a suspected under-reported procedure was found in the HRs, we consulted the CapaCare schedule. If the participant was not posted at that hospital at the time, and another participant with the same given name was, the procedure was not added as an under-reported procedure.

When a procedure in the HRs was registered as a match or a close match with a database entry, that procedure was not registered as a match or a close match for any other database entries for the same participant. The same procedure could be registered as a match or close match for another participant because several participants can be involved in the same procedure.

The personal logbooks (PLs) are divided into an obstetrical part and a surgical part, and obstetric surgery should be logged in both parts. The surgical part is the focus of this study. One participant noted in the interview that he registered obstetric surgery in the obstetric part only. When cross-checking procedures from this participant with the PLs, the obstetric part was used instead of the surgical part for all obstetric surgery.
