# Supplementary material for: Evaluation of a surgical task sharing training programme’s logbook system in Sierra Leone
Source: BMC Med Educ. 2019 Jun 11;19:198. doi: 10.1186/s12909-019-1647-2 (PMC6560768; doi:10.1186/s12909-019-1647-2)
Supplement: Supplementary file 3 — Minor and major procedures. Description of which procedures were defined as minor and major. (DOCX 14 kb) [file 12909_2019_1647_MOESM3_ESM.docx]

**Additional file 3
Minor and major procedures**

**Minor procedures**

Breech extraction

Dilatation and curettage

Induction of labour

Uterus tamponade for postpartum haemorrhage

Repair of cervical/vaginal/perineal tears

Instrumental delivery (vacuum extraction)

Manual placenta removal

Manual vacuum aspiration

POP casts for fractures

Reposition of joint

Skeletal traction

Cleaning and dressing

Chest tube

Evacuation of hematoma

Excision of lipoma

Secondary closure

Suprapubic puncture

Urethral dilation for urethral stricture

Wound suturing

Excision other

Incision and drainage

Necrotectomy

Urethral catheterization

Male circumcision

**Major procedures**

Above-knee amputation

Hand amputation

Below-ankle amputation

Finger amputation

Below-knee amputation

Toe amputation

Uterus prolapse operation

Myomectomy

Craniotomy

Cystectomy

Caesarean section

Repair of bladder injury

Hysterectomy

Repair of ruptured uterus

Salpingectomy for ectopic pregnancy

External fixation

Internal fixation

Osteomyelitis debridement

Appendectomy

Colostomy

Femoral hernia repair

Inguinal hernia repair

Strangulated hernia repair

Umbilical hernia repair

Ventral hernia repair

Laparotomy

Large bowel resection

Orchidectomy

Foreign boody removal

Scrotal hydrocele

Skin-graft

Small bowel resection

Dental extraction

Tubal ligation

Repair of uterine tear

Vesicovaginal fistula repair

**Unspecified**

Obstetrics/gynaecology other

Neurosurgery other

Orthopaedic surgery other

Soft tissue surgery other

Missing
